# Sensor based dataset to assess the impact of urban heat island effect mitigation and indoor thermal comfort via terrace gardens

**DOI:** 10.1016/j.dib.2023.109431

**Published:** 2023-07-17

**Authors:** Girish Visvanathan, Kailas Patil, Yogesh Suryawanshi, Prawit Chumchu

**Affiliations:** a Vishwakarma University, Pune, India; b Kasetsart University, Thailand

**Keywords:** Urban horticulture, Urban farming, Roof top gardening, Thermal insulation, Solar reflective index, Thermal comfort

## Abstract

This dataset contains temperature variations observed on a building terrace that is partially covered with plantations on one side while the other side remains exposed. The study was conducted at a shelter named “Anbagham” in Tamil Nadu, India. Two sets of temperature and humidity sensors were utilized, with one set placed on the external roofs and the other set placed inside the rooms corresponding to these roofs. The analysis spanned over a period of two months, specifically during the hottest period of the year, totaling 66 days, with measurements taken every hour. The provided dataset can be effectively utilized to examine temperature disparities and patterns in the internal environment attributed to the presence or absence of roof gardens. This research and the accompanying dataset have significant implications for various disciplines. They can aid in the planning and design of energy-efficient buildings, assist green building engineers in estimating internal thermal comfort, enable city/urban planners to estimate land surface temperatures, allow botanists to evaluate the impact of foliage on temperature relief, aid civil engineers in proposing green and insulative roof assemblies, and help mechanical engineers estimate reduced cooling loads and corresponding energy savings.

**Specifications Table**

**Table:** 

Subject	Horticulture, Sustainability and environment, Municipal and urban engineering, climatology, environmental engineering, material physics, analytics, geography, instrumentation, plant science- general, signal processing, data science.

Specific subject area	Heat transfer, heat flux, plant foliage, shading effect, solar reflectivity, thermal mass, insulation, thermal comfort, heat flux, urban heat island effect.
Type of data	Table
How the data were acquired	The data collection process involved the use of two distinct sets of sensors. The first set was deployed in the baseline monitoring zone, which represents the area of the terrace without any garden. The purpose of this set was to capture the baseline temperature data. The second set of sensors was installed in the green/heat mitigation monitoring zone, corresponding to the area of the terrace with the garden. The objective here was to record temperature levels specifically in this area to assess the impact of the garden. For the temperature recording, two types of sensors were utilized. The external set comprised of two AM2306 Mounted Temperature and Humidity Sensors, which were positioned to measure the temperature and humidity outside the building. The internal set included two AM2105A Mounted Temperature and Humidity Sensors, placed to monitor the temperature and humidity levels inside the rooms of the building. These sensors were employed to gather comprehensive temperature data from both the external and internal environments of the building.
Data format	Raw
Description of data collection	The data collection focused on evaluating the heat mitigation effects of a rooftop garden, which encompassed approximately 92.90 sq. mt. and consisted of 184 grow bags. Temperature recordings were captured at hourly intervals over a span of 66 days, from 1/04/2023 to 6/06/2023. The analysis aimed to compare the temperature and humidity measurements of the roof area covered with the plantation against the exposed roof area. The dataset encompassed data for two surfaces: the external temperature and humidity readings, as well as the internal temperature and humidity measurements from the underlying areas.
Data source location	Anbagham, Otteri, Puraisaiwakkam, Chennai, Tamil Nadu, India 13°05′37.0"N 80°14′47.5"E Primary data source: Visual Crossing https://www.visualcrossing.com/weather/weather-data-services
Data accessibility	Repository name: Urban Heat Island Mitigation via Rooftop Garden - Sensor based data Data identification number: 10.17632/jbjzvmtfj8.2 Direct URL to data: https://data.mendeley.com/datasets/jbjzvmtfj8/3

## Value of the Data

• Impact of Urban Heat Island Effect: The dataset provides an opportunity to analyze and estimate the reduction in heat ingress and cooling costs through the implementation of rooftop gardens. This has significant implications for addressing the global impact of the urban heat island effect, particularly in European countries where temperatures have exceeded the 40°C mark.
• Enhancing Building Energy Efficiency: Researchers can leverage the dataset to enhance the energy efficiency of buildings by incorporating rooftop gardens, which offer shade and thermal insulation. This contributes to reducing energy consumption and promoting sustainable practices.
• Scientific Studies and Horticulture: The dataset can be utilized in various scientific studies, including horticulture, to explore the potential benefits of green spaces and their impact on the environment. While further data on specific typology of greenery on rooftops and detailed temperature/humidity measurements may be required for in-depth ecosystem analysis, the dataset can still provide valuable insights into general patterns and trends related to promoting flora and fauna growth and potentially improving the air quality index (AQI) in urban areas.
• Food Security Considerations: The dataset's insights have relevance for organizations like the International Food Policy Research Institute (IFPRI) in terms of considering associated advantages, such as the potential mitigation of the heat island effect through rooftop gardens. Although the dataset may not include extensive temperature/humidity data for comprehensive ecosystem analysis, it can still contribute to a broader understanding of sustainable agricultural practices and their potential role in ensuring food security.
• Global Temperature Reduction Estimation: The dataset can be employed in calibrating models to estimate global temperature reduction trends across multiple locations. This aids in understanding the broader impact of implementing rooftop gardens in mitigating climate change.
• Planning and Designing Efficient Buildings: The paper and its dataset serve as valuable resources for master planners, green building engineers, city/urban planners, conservationists, botanists, civil engineers, and other professionals involved in planning and designing efficient buildings. It offers insights into incorporating sustainable and eco-friendly elements into architectural practices.

## 1 Objective

The objective was to evaluate the impact of rooftop gardens on temperature reduction for residents' benefit and explore the wider potential of green interventions in creating sustainable and thermally comfortable urban environments.

## 2 Data Description

The urban heat island effect is a pressing challenge in cities worldwide, driven by factors such as urbanization and the loss of green spaces. Terrace plantations have emerged as a promising solution for mitigating urban heat. These rooftop gardens reduce surface and ambient temperatures, improve thermal comfort, and promote environmental sustainability. Research on the effectiveness of terrace plantations has highlighted their potential in heat mitigation, biodiversity enhancement, and social engagement [1], [2], [3], [4], [5]. Some hydroponics approaches are also used to reduce urban heat and to enhance secondary metabolites [6,8,9]. However, further investigation is needed to optimize design, maintenance, and policy implications. This paper presents a dataset on temperature variations observed in a building terrace with partial plantations, providing valuable insights for urban planning and design decisions.

The data for this study was collected from a rooftop garden project conducted by the Chennai Resilience Center (CRC) at the Anbagham homeless shelter in Otteri, Purasaiwakkam, Chennai. The purpose of the study was to examine the heat mitigation impact of the rooftop garden. The rooftop garden was set up in January 2023, covering an area of approximately 92.90sq. mt. It consisted of 185 mobile vegetable garden kits. The garden was located along the eastern wall of the building and was well established by the time measuring instruments were installed in April 2023. To measure temperature and humidity, two sets of sensors were installed. The first set recorded baseline data from the area of the terrace without the garden (baseline monitoring zone), while the second set recorded data from the area with the garden (green/heat mitigation monitoring zone). The sensors used were cased (IP67 rated) sensors for outdoor measurements and exposed sensors for internal regions. The precise locations of the four installed sensors were external baseline sensor, internal baseline sensor, external green roof sensor, internal green roof sensor. In addition to the installed sensors, secondary weather data for the Otteri neighborhood was sourced from Visual Crossing to provide a larger context for the study. The dataset available in the excel sheet named “UHIE Mitigation roof garden sensor data” which contains hourly data collected from sensors for the following parameters: Dry-bulb temperature (°C) (Data collected from Visual Crossing), External relative humidity (%), External temperature in the garden area, External relative humidity in the garden area, Internal temperature in the garden area, Internal relative humidity in the garden area, External temperature in the exposed area, External relative humidity in the exposed area, Internal temperature in the exposed area, and Internal relative humidity in the exposed area. The data spans from April 1st, 2023, to June 6th, 2023, covering a period of 66 days.

## 3 Experimental Design, Materials and Methods

**Fig. 1:**
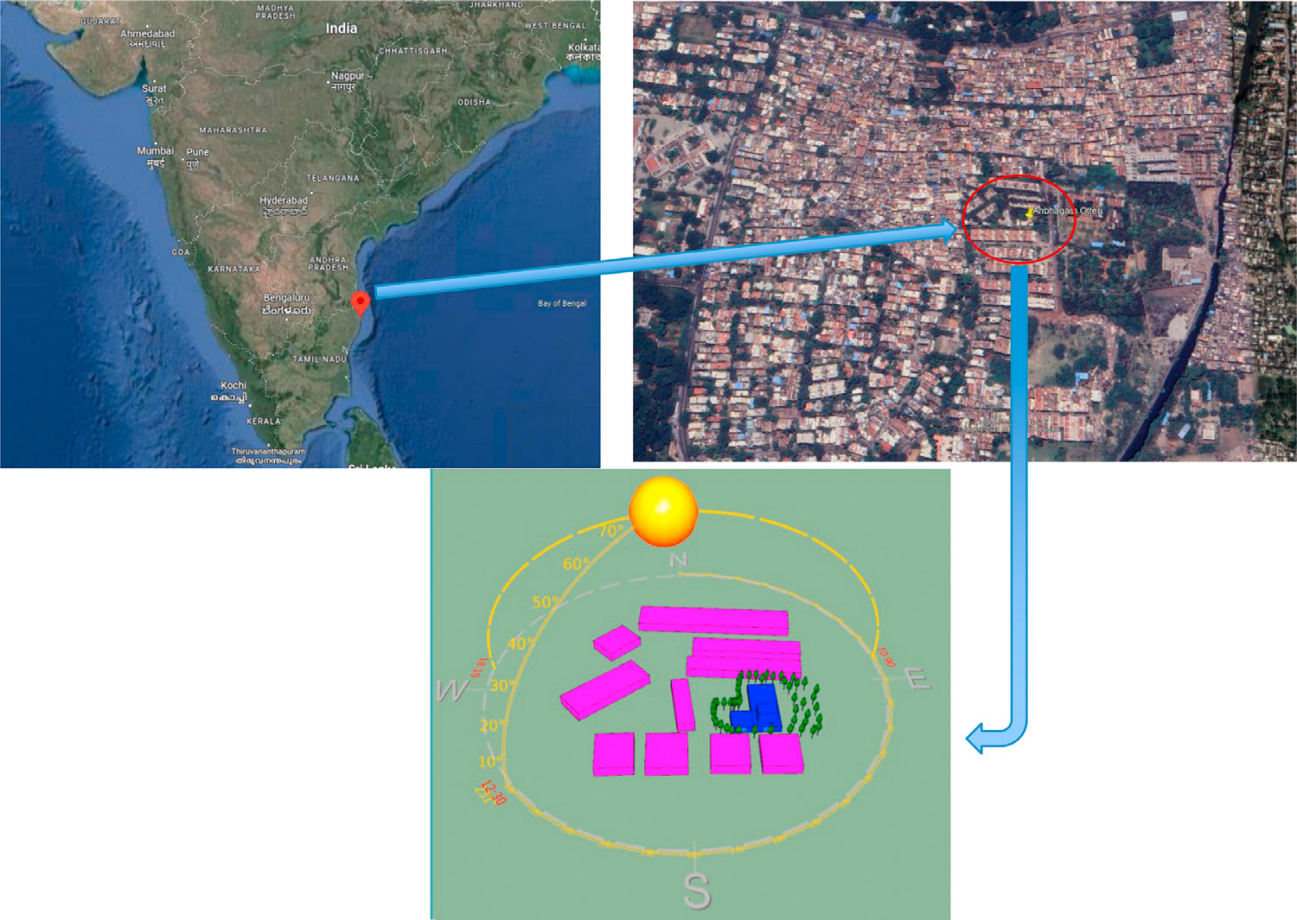
Site location in satellite (top) and simulated (bottom) images.

In January 2023, Chennai Resilience Center (CRC) helped a homeless shelter for women with psychosocial challenges set up a ∼92.90 sq mt rooftop garden using around 185 mobile vegetables garden kits. This shelter, called Anbagham, is located in Otteri, a neighborhood in Purasaiwakkam, in the Western part of Chennai (Fig. 1). The study was conducted in a facility for differently abled and mentally challenged individuals who spend time indoors. Rooftop gardens were implemented to provide temperature relief, engage residents in gardening, and produce edible crops. CRC chose this site for conducting a case study to examine the heat mitigation impact of a rooftop garden.

At the time of installation of the measuring instruments (April 2023) the terrace garden located along the eastern wall of the building with 184 grow bags covering an approximate area of 92.90 sq. mtwas already well established, ensuring an adequate green density. To prevent water seepage from the planter bags and to prevent the area from acting as a thermal mass, empty white bags made of polypropylene polymer (plastic) were used to cover the planter bag area and the exposed eastern wall stretch.

Two sets of temperature and humidity sensors were installed which included, cased (IP67 rated) sensors for outdoor and exposed sensors for internal regions. Both senseors Range and accurancy arre given in Table 1.

**Table 1:** Details of sensors used to measure humidity and temperature of terrace surface.

Parameters	Details
Typical accuracy (% RH)	± 2
Operating range (% RH)	0 to 99.9
Typical accuracy (°C)	0.5
Operating range (°C)	-40 ∼ 80
Interface	1 – Wire
Supply Voltage (V)	3.3 ∼ 5.5

**Fig. 2:**
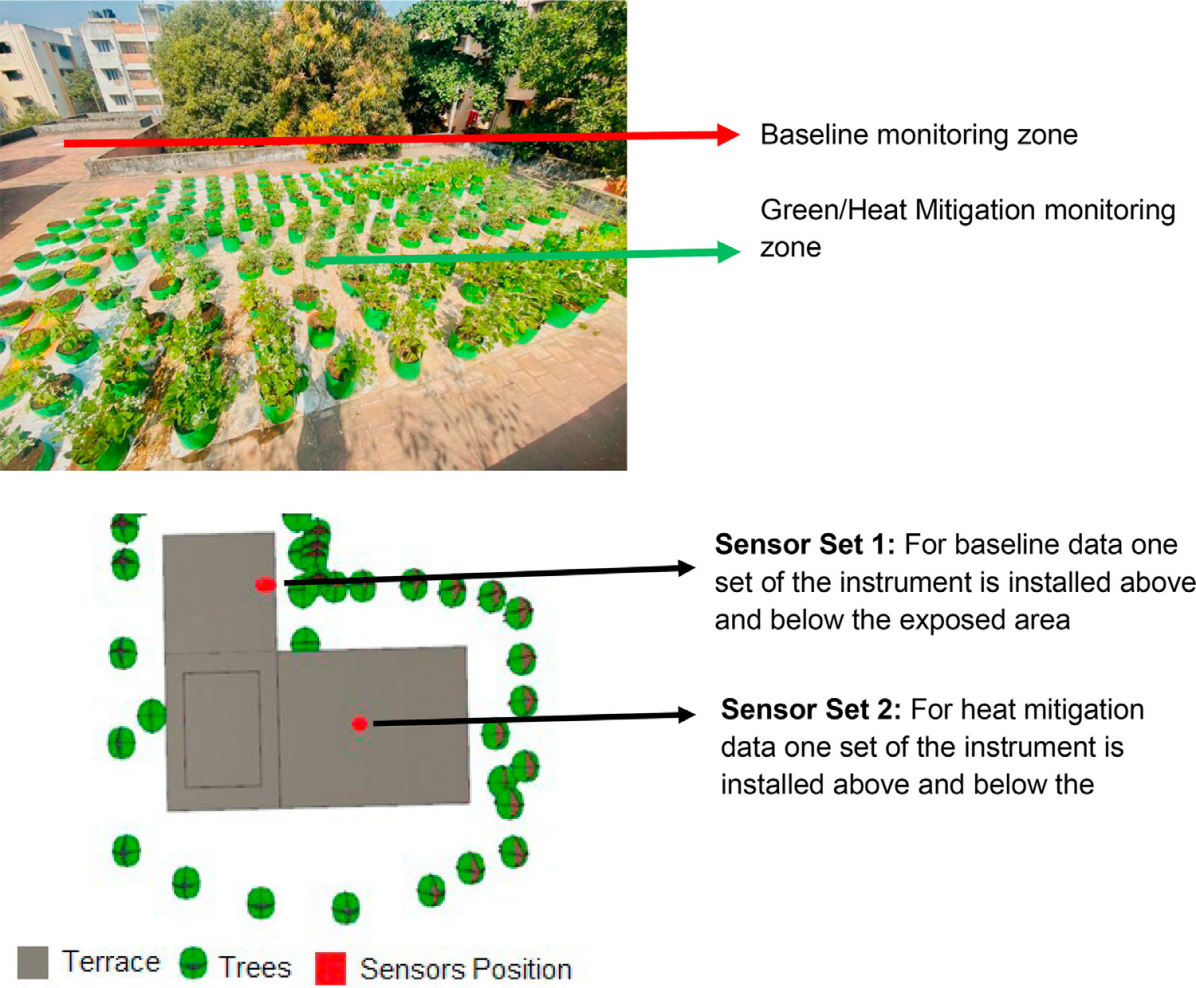
Baseline and garden sites.

One set of sensors were used to record baseline data from the area of the terrace where there is no garden (the baseline monitoring zone) (41.80 sq. mt.) and the second set was Installed to record temperature in the area with the garden (the green/heat mitigation monitoring zone) (Fig. 2).

**Fig. 3:**
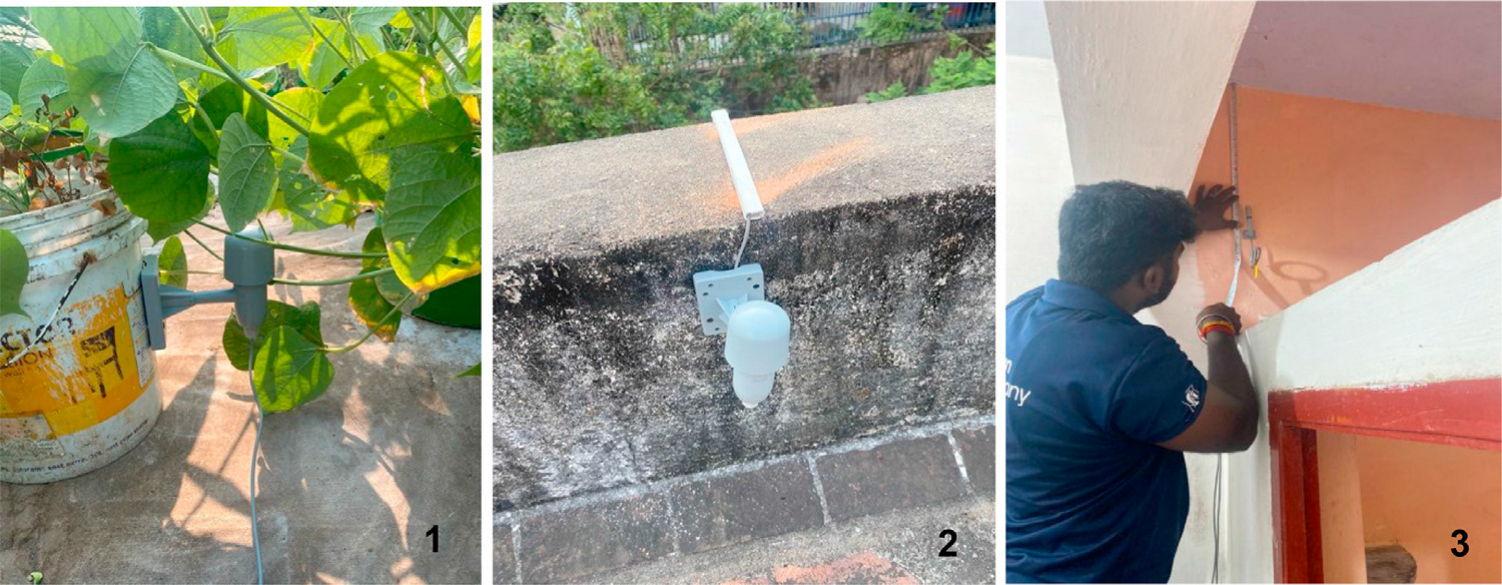
A sensor in the garden area (1), a sensor in the exposed area (2) and a sensor in one of the internal spaces (3).

The precise location of the 4 installed sensors were as follows (Fig. 3):

• The Sensor measuring external baseline data was set up in the Southwest corner of the terrace in the exposed portion of the terrace.
• The Sensor measuring internal baseline data was set up on the restroom ceiling, in the Southwest corner, below the external sensor.
• The external sensor measuring data from the green roof was installed amidst the planter bags, close to the parapet wall.
• A corresponding internal sensor to measure impact of the green terrace was installed on the ceiling of the store room directly below the planter bags.

Apart from our own sensors, we also relied on secondary weather data for the larger Otteri neighborhood, sourced from Visual Crossing.

A simple system was used to process and calibrate the data captured through the sensors. A microcontroller programmed based on guidance from the sensor manufacturer was used to read the data signals. The controller interpreted these signals and processed the data using a web application called MATLAB. It then shared the data points via WIFI to a cloud application which was previously configured for this task. As such the sensors themselves do not need calibration unless a discrepancy or mismatch was observed in the collected data. However, we did calibrate the sensors to the extent of installing a new Wi-Fi dongle to ensure that reporting intervals of data were for the same time stamps. To ensure accuracy of data collected, the team used a handheld temperature measurement device to intermittently check the temperature and compare it with the recorded data and calibrate if needed. Data from the sensors were reported on an hourly basis collected for the period of April 1^st^ 2023 to June 6^th^, 2023 (66 days). The following steps were carried out the assess data recorded during this period:

• Extreme measurements of low and high temperature (out of range data) were removed to ensure the results were not skewed.
• This data was then compared with the area specific weather data (Otteri weather data) to estimate the variance between weather data, external and internal temperature.
• This tabulation was then used to calculate the following:
  - The heat retained by surfaces by comparing weather data with external surface temperature of the exposed terrace space and exposed space with garden over time.
  - The temperatures inside the building and on the corresponding surface space above.
  - Temperatures in the room below the exposed surface area and the terrace garden that would indicate the impact of the garden.

The Dataset provides accurate, objective, and real-time measurements, allowing for long-term monitoring, comparative analysis, and validation of design interventions. The dataset supports evidence-based decision-making, policy formulation, and the development of sustainable urban environments. The dataset provides the information of external relative humidity of the garden (Ext RH Garden), internal temperature of the garden (Internal Temp Garden), internal relative humidity of the garden (Internal RH Garden), external temperature of the exposed area (Ext Temp Exposed), external relative humidity of the exposed area (Ext RH Exposed), internal temperature of the exposed area (Internal Temp Exposed), internal relative humidity of the exposed area (Internal RH Exposed). The Temperature and humidity dataset is uploaded on Mendeley data [7].

The collected data from the sensors were processed and calibrated using a microcontroller programmed based on guidance from the sensor manufacturer. The data signals were interpreted and processed using MATLAB, a web application. The data points were then transmitted via Wi-Fi to a cloud application for storage and analysis. The sensors themselves did not require calibration unless a discrepancy or mismatch in the collected data was observed. However, a new Wi-Fi dongle was installed to ensure consistent reporting intervals of data. To ensure the accuracy of the collected data, handheld temperature measurement devices were used intermittently to cross-check the temperature readings and calibrate the sensors if necessary. Data were reported on an hourly basis for the period from April 1st, 2023, to June 6th, 2023, totaling 66 days. Before analysis, extreme low and high temperature measurements that fell outside the expected range were removed to ensure the integrity of the results.

The data collected from the sensors were compared with the area-specific weather data for Otteri to estimate the variance between weather data, external and internal temperatures. This tabulation was then used to calculate the heat retained by surfaces, the temperatures inside the building and on the corresponding surface space, and the temperatures in the room below the exposed surface area and the terrace garden, which indicated the impact of the garden on heat mitigation.

## Ethics Statements

The collection of temperature and humidity data from the building terrace with rooftop gardens and the corresponding exposed areas followed ethical protocols. One of the author's building and terrace area was used in the study and data collection. The data collection process prioritized the privacy and confidentiality of the participants, with measures taken to anonymize and secure the collected data. We did not conducted the human or animal studies. We have purchased the professional subscription of the Visual Crossing for collecting weather data of particular area. We did not need a permission to use the primary data from Visual Crossing.

## Credit Author Statement

**Girish R. Visvanathan**: Conceptualization, Methodology, Data Collection, and Calibration **Kailas Patil:** Data curation, Writing- Original draft preparation, supervision. **Yogesh Suryawanshi:** Writing- Original draft preparation **Prawit Chumchu:** Visualization, Investigation reviewing.

## Data Availability

Urban Heat Island Mitigation via Rooftop Garden - Sensor based data (Original data) (Mendeley Data).
